# Lightweight Hot-Spot Fault Detection Model of Photovoltaic Panels in UAV Remote-Sensing Image

**DOI:** 10.3390/s22124617

**Published:** 2022-06-18

**Authors:** Qiuping Zheng, Jinming Ma, Minghui Liu, Yuchen Liu, Yanxiang Li, Gang Shi

**Affiliations:** College of Information Science and Engineering, Xinjiang University, Urumqi 830046, China; zhengqiuping@stu.xju.edu.cn (Q.Z.); majinming@stu.xju.edu.cn (J.M.); lmh95810@stu.xju.edu.cn (M.L.); liuyuchen@stu.xju.edu.cn (Y.L.); liyanxiang@stu.xju.edu.cn (Y.L.)

**Keywords:** photovoltaic panels, hot spot, failure detection, neural network

## Abstract

Photovoltaic panels exposed to harsh environments such as mountains and deserts (e.g., the Gobi desert) for a long time are prone to hot-spot failures, which can affect power generation efficiency and even cause fires. The existing hot-spot fault detection methods of photovoltaic panels cannot adequately complete the real-time detection task; hence, a detection model considering both detection accuracy and speed is proposed. In this paper, the feature extraction part of YOLOv5 is replaced by the more lightweight Focus structure and the basic unit of ShuffleNetv2, and then the original feature fusion method is simplified. Considering that there is no publicly available infrared photovoltaic panel image dataset, this paper generates an infrared photovoltaic image dataset through frame extraction processing and manual annotation of a publicly available video. Consequently, the number of parameters of the model was 3.71 M, mAP was 98.1%, and detection speed was 49 f/s. A comprehensive comparison of the accuracy, detection speed, and model parameters of each model showed that the indicators of the new model are superior to other detection models; thus, the new model is more suitable to be deployed on the UAV platform for real-time photovoltaic panel hot-spot fault detection.

## 1. Introduction

In July 2021, SolarPower Europe issued The Global Market Outlook Report for 2021 to 2025 [1]. In 2020, the net installed capacity of natural gas, hydropower, photoelectric, wind, and other renewable energy sources was 138.2 GW. Photovoltaic panels were the power generation technology with the highest net installed capacity, accounting for 39%. The report predicts that, by 2025, the world’s grid-connected photovoltaic capacity will reach 1553 GW in the worst-case scenario, 1604 GW in the middle scenario, and 2147 GW in the best-case scenario. The development of photovoltaic power stations will provide a way for the world to use renewable resources. Compared with traditional fossil fuels such as oil and coal, photovoltaic panels have no noise generation and no pollution in the power generation process, and they have the advantages of safety and sustainability [2].

Photovoltaic panels exposed to the harsh outdoor environment for a long time are susceptible to thunderstorms, ultraviolet radiation, and thermal cycling, resulting in cracking and damage [3,4]. These failures will lead to local heating of photovoltaic panels, resulting in hot-spot failure. Hot spots consume power generated in other areas of the panel, reducing power generation efficiency and releasing heat. As the temperature rises, it can melt solder joints on the panels, damaging them and causing a fire [5,6].

The research on hot-spot fault detection of photovoltaic panels can be roughly divided into two directions: using the electrical characteristics of photovoltaic panels and using the infrared image characteristics of photovoltaic panels [7,8].

When using the electrical characteristics of photovoltaic panels for hot-spot fault detection, it is necessary to obtain the electrical characteristics of photovoltaic panels, such as voltage and current, and then input these characteristics into intelligent algorithms or mathematical–statistical models for analysis [9,10,11]. Another common approach is to use temperature and pixel information from images of infrared photovoltaic panels. Noncontact detection helps maintain the performance of photovoltaic panels, thus prolonging the service life of the equipment and generating greater economic returns [12]. With the advantages of low cost, high efficiency, wide field of vision, and no contact, UAVs are widely used in the detection of various targets [13,14,15]. This also provides a new solution to the repetitive and tedious hot-spot fault detection task of photovoltaic power stations [16]. Deep learning can better solve high-dimensional, redundant, and high-noise big data problems without the need for manual design features and input empirical knowledge [17]. When detecting infrared photovoltaic panel images taken by UAV, the lightweight deep learning method can not only improve the robustness and accuracy of hotspot detection in a complex environment but also speed up detection and reduce resource consumption.

The main contributions of this study can be summarized as follows:

(i) A new lightweight fault detection technique based on deep learning is proposed to solve and fill the gaps proposed in the literature survey.
(ii) An infrared photovoltaic panel image dataset was made by integrating and processing the previous public data.
(iii) The original feature extraction and feature fusion methods of the YOLOv5 were optimized to improve the detection accuracy of photovoltaic panels and hot spots. S-YOLOv5 has good performance in terms of detection accuracy, number of parameters, and detection speed.

The paper is organized as follows: Section 2 describes the related work, and Section 3 introduces the proposed methods. Section 4 presents the testing results and provides a comparison with existing methods. In Section 5, a summary of the work and prospects for future work are given.

## 2. Related Works

### 2.1. Hot-Spot Fault Detection Based on the Electrical Characteristics of Photovoltaic Panels

Harrou et al. [18] calculated the difference between the theoretical output value and the actual output value of photovoltaic panels, and then input the difference into the improved K-nearest neighbor (KNN) algorithm. The exponential weighted moving average (EWMA) based on KNN achieves good performance in hot-spot fault detection. Hariharan et al. [19] proposed a method to detect photovoltaic panel faults and different degrees of photovoltaic panel shielding by using voltage, current, and irradiance parameters of photovoltaic modules. Dhimish et al. [20] proposed a method based on numerical analysis, which sets the threshold of photovoltaic panel failure by analyzing the relationship between the theoretical value and the output value in different states. The above methods depend on the accuracy of a simulation model. Photovoltaic panels that run outdoors for a long time are prone to aging; therefore, the simulation model needs to be constantly updated to reduce the output deviation from the actual photovoltaic panels. Winston et al. [21] used the percentage of power loss (PPL), open-circuit voltage (V_OC_), short-circuit current (I_SC_), irradiance (I_RR_), panel temperature, and internal impedance (Z) parameters. The hot spots of photovoltaic panels were detected by using a feedforward backpropagation neural network and support vector machine (SVM). The average accuracy of the feedforward backpropagation neural network was 87%. The accuracy of the SVM was 99%. This method requires the photovoltaic panels to stop working, and it takes a long period to obtain the *I–V* curve; hence, real-time diagnosis is difficult. Rossi et al. [22] not only verified the dramatic temperature rise of PV cells under shading through experimental data but also proposed a hot-spot detection scheme. In general, these methods require building physical circuits around photovoltaic panels and installing sensors to obtain electrical characteristics [23]. Some fault detection methods also need to stop the work of photovoltaic panels, which prevents achieving real-time detection, thus affecting the efficiency of power generation.

### 2.2. Hot-Spot Fault Detection Based on the Infrared Image Features of Photovoltaic Panels

In a small number of photovoltaic panel detection tasks, many scholars are still using infrared photovoltaic panel images taken on the ground for hot-spot fault detection. Hwang et al. [24] converted the image format from RGB to HSV, and then used the gamma correction function to enhance the S and V values of red pixels, before finally sending them into the convolutional neural network to learn hot-spot fault features. Ali et al. [25] used RGB, texture, directional gradient histogram (HOG), and local binary mode (LBP) features to form a new hybrid feature vector by data fusion. Then, SVM was used to classify the thermal images of photovoltaic panels. This traditional detection method is based on manual features and shallow trainable architecture, which combines a large number of low-level image features with high-level semantic information from the target detector and scene classifier to build a complex system. However, this method has low detection speed and low detection performance. For super-large photovoltaic power stations, if photovoltaic panels can only be detected block by block, this will undoubtedly reduce the detection efficiency and increase the cost of detection.

UAVs carrying infrared cameras can collect data quickly, cheaply, and efficiently, reducing labor costs. To efficiently complete the hot-spot fault detection of large photovoltaic power stations, many scholars also shifted their research focus to infrared photovoltaic images taken by the UAV. Pierdicca et al. [26] sent infrared photovoltaic panel images collected by UAV into the Visual Geometry Group Network (VGG) to learn the hot-spot fault features. However, the number of VGG-16 parameters is about 138 million, which means that the number of model parameters is too large and requires a lot of computing resources. Herraiz et al. [27] combined two region-based convolutional neural networks to generate a robust detection method and combined photovoltaic panel images with visible images of thermal imaging to provide more hot-spot fault information. Aghaei et al. [28] used the Harris corner detection algorithm for image mosaic and the edge detection method for feature extraction to realize the statistics of fault-free photovoltaic modules and failure photovoltaic modules. Dunderdale et al. [29] proposed a hot-spot fault detection model combining VGG-16 and MobileNet, using scale-invariant feature transform (SIFT) and a random forest classifier to identify failure types. 

**Figure 1:**
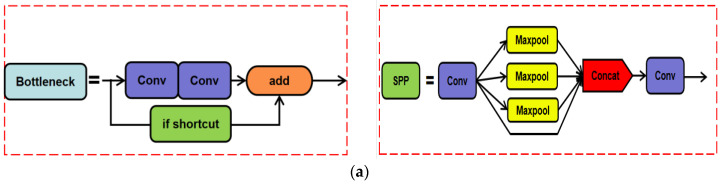

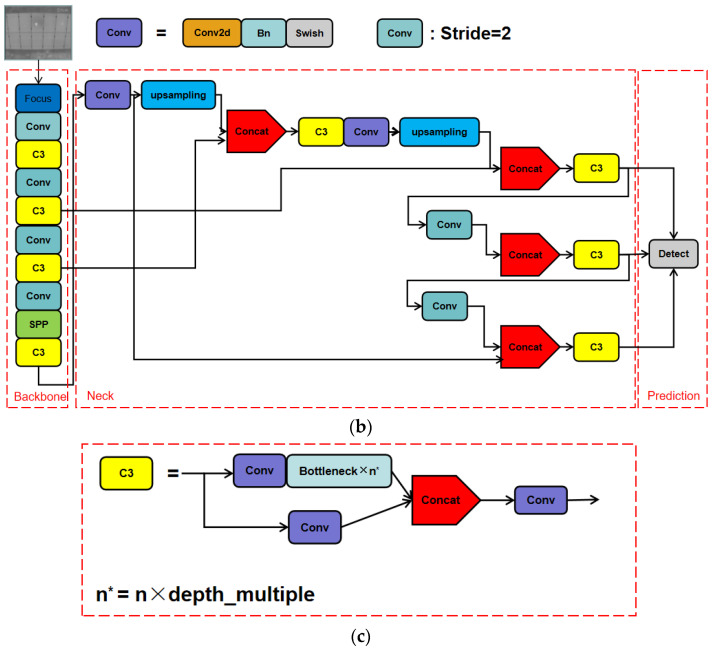
(**a**) Architecture of Bottleneck and SPP. (**b**) Architecture of YOLOv5 network. (**c**) Architecture of C3.

Traditional digital image processing algorithms need prior knowledge and are not robust. YOLOv5 [30] is a single-stage target detection algorithm, whose detection accuracy and speed are better than those of many algorithms. Figure 1 shows the network structure of YOLOv5. Therefore, this paper used YOLOv5 as the overall framework to achieve hot-spot fault detection in infrared photovoltaic panel images. The backbone network of the latest version of YOLOv5 uses Focus, Conv, C3, and SPP, while the neck part adopts the combined structure of the feature pyramid network (FPN) [31] and path aggregation network (PAN) [32].

## 3. Proposed Method

**Figure 2:**
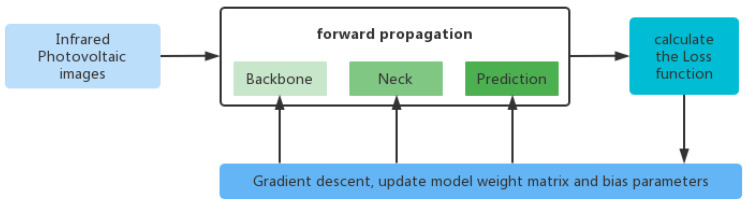
The training process of S-YOLOv5.

S-YOLOv5 divides the training process of hot-spot fault detection into four steps, as shown in Figure 2. The first step is adaptive scaling and normalization of the image. The second step is to obtain the position information of the prediction box through forward propagation. Forward propagation includes feature extraction from the backbone, feature fusion of the neck, and prediction. The third step uses the loss function to calculate the difference between the prediction box and the ground truth. The fourth step uses the gradient descent method to update the weight matrix and bias parameters in the forward propagation to reduce the loss value. 

**Figure 3:**
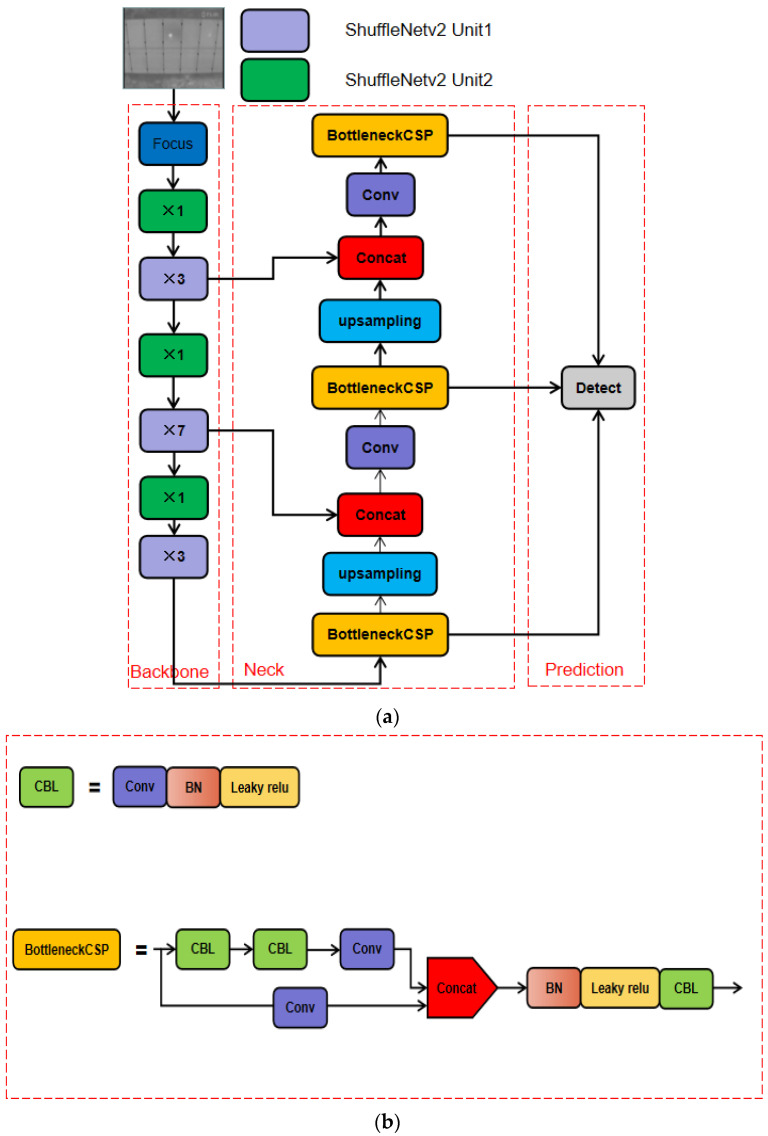
(**a**) Architecture of S-YOLOv5 network. (**b**) Architecture of BottleneckCSP.

The network structure of S-YOLOv5 is shown in Figure 3. The infrared camera carried by the UAV has different resolutions, resulting in different image sizes. Before the image is input into the network, adaptive scaling is required. Adaptive scaling obtains the width and height information of the input image. The shrinkage in width and height can be calculated separately, taking the minimum. Finally, the shrinkage rate is used to calculate the pixels that need to be filled in the original image.

**Figure 4:**
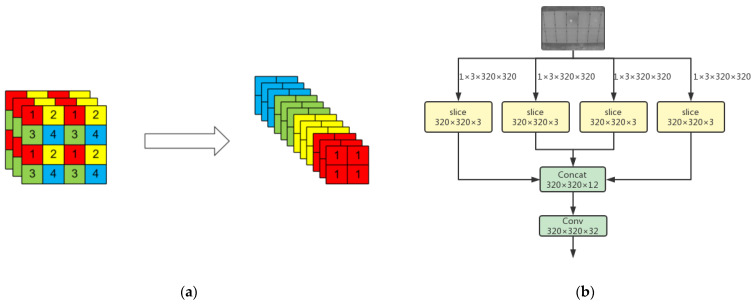
(**a**) Focus slicing process. (**b**) Structure of Focus module.

The backbone consists of Focus and the base unit of ShuffleNetv2 [33]. The number after × indicates the number of modules. Focus is located in the top layer of the backbone and can sample the images of the input neural network. Figure 4 shows the slicing process of Focus. The input image initially has only three channels, as shown in Figure 4a. Focus separates elements of each layer feature map to form four new feature maps. As a result, the number of channels is expanded from three to 12. After the Focus operation, four groups of down-sampling feature images of 320 × 320 × 3 were generated, as shown in Figure 4b. Then, the Concat operation was performed on the 320 × 320 × 3 feature images to obtain the 320 × 320 × 12 feature map. Finally, the 320 × 320 × 32 feature image was generated through the Conv operation using 32 convolution kernels.

**Figure 5:**
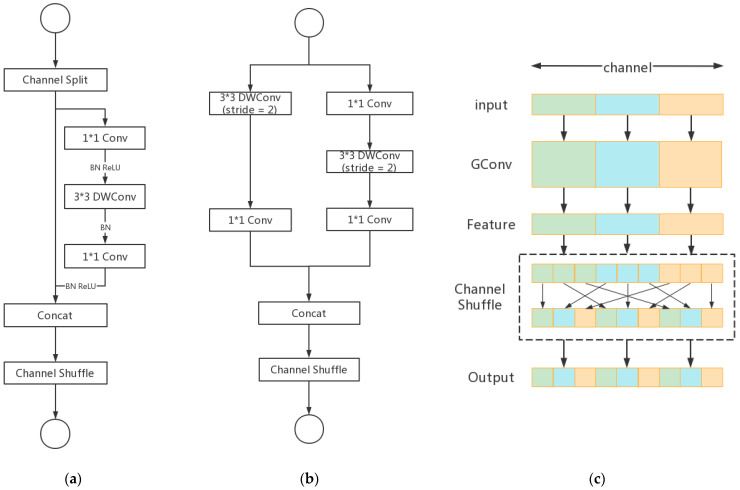
(**a**) ShuffleNetv2 unit1. (**b**) ShuffleNetv2 unit2. (**c**) Channel shuffle.

Figure 5a,b show two ShuffleNetv2 basic units. ShuffleNetv2 unit1 splits channels into two parts; one part directly passes downward, and the other part calculates backward. Concat effectively avoids element-wise operation and reduces time consumption. ShuffleNetv2 unit2 is the spatial down-sampling unit. DWConv can be understood as the fusion of channel-by-channel convolution and point-by-point convolution. One-channel convolution refers to the convolution operation based on maintaining the same spatial channel. Point-by-point convolution is a deep convolution over a space channel.

Figure 5c shows how the channel shuffle works. After the group convolution operation of the input features of different channels, the feature information of the channel is still output. After the output features are rearranged, the original channels are mixed with the feature information of other channels to enhance the information communication between channels. If only the splicing operation is carried out, the feature information after convolution cannot be better used, which also affects the detection accuracy of the model. Therefore, the basic unit of ShuffleNetv2 performs a channel shuffle operation on the two parts of the channel that were previously spelled, which reduces the computational complexity of the model and greatly improves the computational efficiency.

The neck part of S-Yolov5 is a feature fusion structure, which effectively makes use of high-level semantic information and low-level high-resolution information. In this paper, the features extracted from the second and fourth layers in the backbone were fused with the network layer in the neck by FPN. Compared with the featured image pyramid method which generates the corresponding scale feature map directly from different scale images, this method effectively reduces the computation, memory resources, and reasoning time of the model. Then, the three BottleneckCSP structures output the feature maps of three scales for target prediction.

The loss function of S-YOLOv5 consists of classification loss, localization loss, and confidence loss.

(1) $$\mathrm{Loss}=l_{\mathrm{cls}}+l_{\mathrm{obj}}+l_{\mathrm{box}}.$$

BCE (binary cross-entropy) is used in the classification loss function, and $I_{ij}^{\mathrm{object}}$ determines whether it is a positive sample; its value is either 0 or 1. ${\hat{P}}_{i}^{j}$ is the sample value. $P_{i}^{j}$ is the predicted value. The calculation method is as follows:

(2) $$l_{\mathrm{cls}}=-\sum_{i=0}^{S^{2}}I_{ij}^{\mathrm{object}}\sum_{c\in classes}\left({\hat{P}}_{i}^{j}\left(c\right)\log\left(P_{i}^{j}\left(c\right)\right)+\left(1-{\hat{P}}_{i}^{j}\left(c\right)\right)\;\log\left(1-P_{i}^{j}\left(c\right)\right)\right).$$

BEC loss is used in the confidence loss function, $\lambda_{\mathrm{object}}$ is the weight coefficient of the positive sample. $\lambda_{\mathrm{noobject}}$ is the weight coefficient of the negative sample. $I_{ij}^{\mathrm{noobject}}$ determines whether it is a negative sample; its value is either 0 or 1. $\overset{S^{2}}{\underset{i=0}{\Sigma }}\overset{B}{\underset{j=0}{\Sigma }}$ indicates the traversal of all prediction boxes. The calculation method is as follows:

(3) $$\begin{array}{ll} l_{\mathrm{obj}}= & -\lambda_{\mathrm{object}}\sum_{i=0}^{S^{2}}\sum_{j=0}^{B}I_{ij}^{\mathrm{object}}\left({\hat{C}}_{i}^{j}\log C_{i}^{j}+\left(1-{\hat{C}}_{i}^{j}\right)\;\log\left(1-C_{i}^{j}\right)\right)\\ & -\lambda_{\mathrm{noobject}}\sum_{i=0}^{S^{2}}\sum_{j=0}^{B}I_{ij}^{\mathrm{noobject}}\left({\hat{C}}_{i}^{j}\log C_{i}^{j}+\left(1-{\hat{C}}_{i}^{j}\right)\;\log\left(1-C_{i}^{j}\right)\right) \end{array}$$

CIOU loss is used in the localization loss function, and the calculation method is as follows:

(4) $$l_{\mathrm{box}}=1-IoU+\frac{\rho^{2}\left(b,b^{gt}\right)}{c^{2}}+\alpha \nu \;\nu =\frac{4}{\pi^{2}}{\left(\arctan\frac{\varpi^{gt}}{h^{gt}}-\arctan\frac{\omega }{h}\right)}^{2},\;\alpha =\frac{\nu }{\left(1-IoU\right)+\nu }$$

where *b* is the center point of the prediction box, *b^gt^* is the center point of the object box, *ρ* is the Euclidean distance, *c* represents the diagonal distance of the smallest outer rectangle formed between the intersecting prediction box and the object box, α is a weight coefficient, and *ν* represents the consistency of the aspect ratio.

## 4. Experiments

To demonstrate the gap between the proposed method and the original baseline model, we performed an ablation study on the improved feature extraction and feature fusion methods. Compared with other mainstream object detection algorithms, the improved algorithm in this paper had better performance in terms of detection accuracy, number of model parameters, and detection speed, which also proves that the improved algorithm is effective in photovoltaic panel hot-spot detection. The training and testing tasks were carried out on a computer equipped with an AMD Ryzen7-5800H processor, 16 GB of RAM, and a GeForce RTX 3060 graphical processing unit (6 GB global memory), running the Windows10 operating system.

After a preliminary experiment, the initial learning rate was fixed at 0.01. We used a weight decay of 0.0005 and a momentum of 0.937. In the training phase, the size of the input image was adjusted to 320 × 320. The batch size of the model training was set to 32. All the comparative experiments were trained for 150 periods.

### 4.1. Infrared Photovoltaic Images Dataset

**Figure 6:**
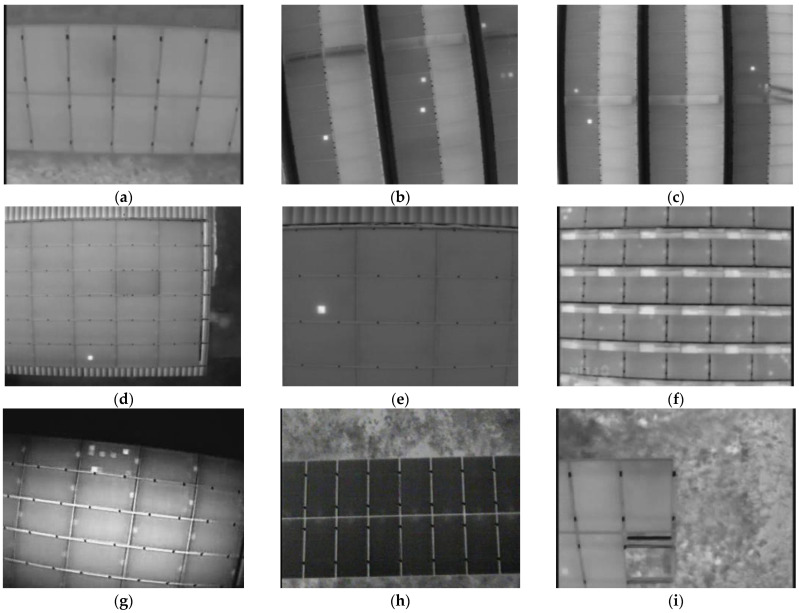
Partial infrared photovoltaic image dataset. (**a**) The UAV took photos along the horizontal direction of the photovoltaic panel. (**b**) The UAV took photos along the tilt angle of the photovoltaic panel. (**c**) The UAV took photos along the vertical direction of the photovoltaic panel. (**d**) Long-distance shooting. (**e**) Close-range shooting. (**a**,**d**–**g**) are different types of photovoltaic panels. (**h**,**i**) are different photovoltaic panel placement methods.

The video used in this experiment was made public by Vincenzo and Antonio. This paper extracted frames from the collected videos into pictures [34,35]. Figure 6 shows some typical infrared photovoltaic panel images. The flight path of the UAV included horizontal, rotational, and vertical directions. The photovoltaic panel videos were captured by three thermal imaging cameras with different resolutions of 640 × 480, 336 × 256, and 320 × 240. The UAV shot images at different heights, diversifying the area occupied by photovoltaic panels in the image. The angle between the photovoltaic panel and the ground was diverse, resulting in different inclinations. The dataset contains photovoltaic panels of different varieties, sizes, colors, and models.

The infrared photovoltaic image dataset was divided into a training set, validation set, and test set, with a ratio of 3:1:1. The dataset included two targets: panels and hot spots. There were 3360 pictures in the training set, 1120 pictures in the validation set, and 1120 pictures in the test set. In the training set, there were 75,102 photovoltaic panels and 4990 hot spots. In the validation set, there were 24,760 photovoltaic panels and 1560 hot spots. In the test set, there were 24,860 photovoltaic panels and 1520 hot spots. Table 1 describes the dataset in detail.

**Figure 7:**
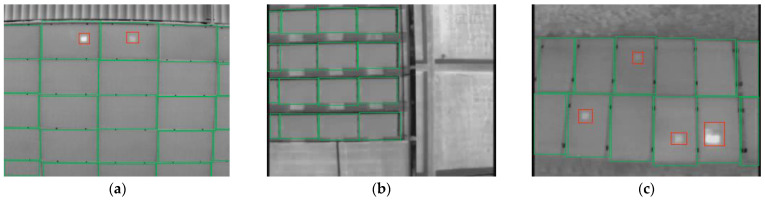
Pictures of the infrared photovoltaic panels after manual annotation: (**a**) two hot spots and 20 photovoltaic panels; (**b**) 16 photovoltaic panels; (**c**) 12 photovoltaic panels and four hot spots.

LabelImg [36], an open-source image annotation tool, was used to annotate infrared photovoltaic images, and then generate annotation data. Rectangular boxes were used to label photovoltaic panels and hot spots in infrared images. In Figure 7, the red frames indicate hot spots, and the green frames indicate photovoltaic panels.

### 4.2. Evaluation Metrics

To comprehensively evaluate the applicability of the photovoltaic panel hot-spot fault detection algorithms, precision, recall, mAP (mean average precision), and FPS (frames per second) were used in this paper.

In the object detection algorithm, there is an intersection ratio between the real frame region of a target and its prediction box region. Assuming that the area of the real box is A and the area of the prediction box is B, the cross union ratio is A ∩ B divided by A ∪ B. When the two boxes overlap completely, the cross union ratio is equal to 1; when the two boxes have no intersecting area, the cross union ratio is equal to 0, and the cross union ratio is set to the threshold α. TP represents the number of predictive boxes whose union ratio is greater than α. FP represents the number of predictive boxes whose union ratio is less than α. FN represents the number of objects in the image that are targeted but not detected. TN refers to the number of negative samples that are negative but not detected. There are countless cases that are not considered. TP, FN, and FP values can be used to calculate other evaluation indices to measure the effect of the object detection algorithm.

Precision refers to the proportion of correctly identified samples to all identified samples.

(5) Precision=TP/(TP+FP).

Recall indicates the proportion of the number of samples correctly identified as positive to all positive samples.

(6) Recall=TP/(TP+FN).

AP effectively avoids the incompleteness of precision and recall in evaluating model performance. AP is the average of precision in the smoothed PR (precision–recall) curve area formed by precision and recall, indicating the precision of a category in the target to be detected.

(7) $$\mathrm{AP}=\int_{0}^{1}\mathrm{Precision}\left(\mathrm{Recall}\right)d\mathrm{Recall}.$$

When the dataset contains multiple types of targets, mAP is used to represent the mean value of each type of target.

(8) $$\mathrm{mAP}=\frac{1}{N}\sum_{i=1}^{N}{\mathrm{AP}}_{i},$$

where *N* is the category of the target in the datasets.

FPS refers to the number of images that can be processed per unit of time. With the same hardware resources, a larger FPS of the object detection algorithm results in more images being detected per unit time and better real-time performance.

### 4.3. Ablation Study

In this part, the impact of the proposed method on the control experiment based on the infrared photovoltaic image dataset is discussed.

The YOLOv5 algorithm was used as the baseline network to explore the influence of Focus, ShuffleNetv2, and FPN on the model. The + in Table 2 represents a hybrid improvement of the module. The precision, recall, and mAP of the baseline network were 93.7%, 92%, and 94.4%, respectively. When the backbone of the network was directly replaced by the lightweight ShuffleNetv2, the number of model parameters was greatly reduced, but the mAP was reduced by 1.4%. When Focus replaced the ordinary convolutional layer in the first layer of ShuffleNetv2, more image features were entered into the network, and mAP improved by 2.6% compared with the baseline. By using the improved FPN feature fusion method, the number of model parameters was reduced by 13.6% while the mAP was maintained. Compared with the original baseline model, the precision, recall, and mAP were improved by 2.3%, 5.2%, and 3.7% respectively, while the model parameters were reduced by 3.35 M.

**Figure 8:**
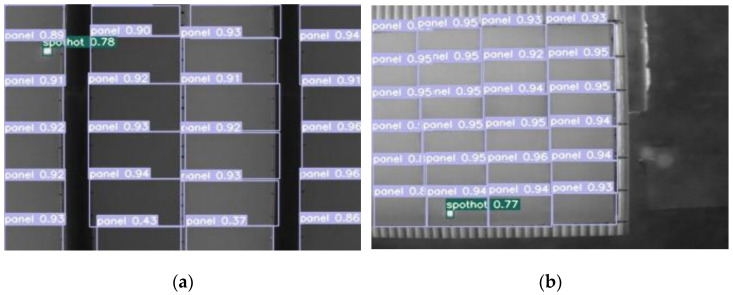

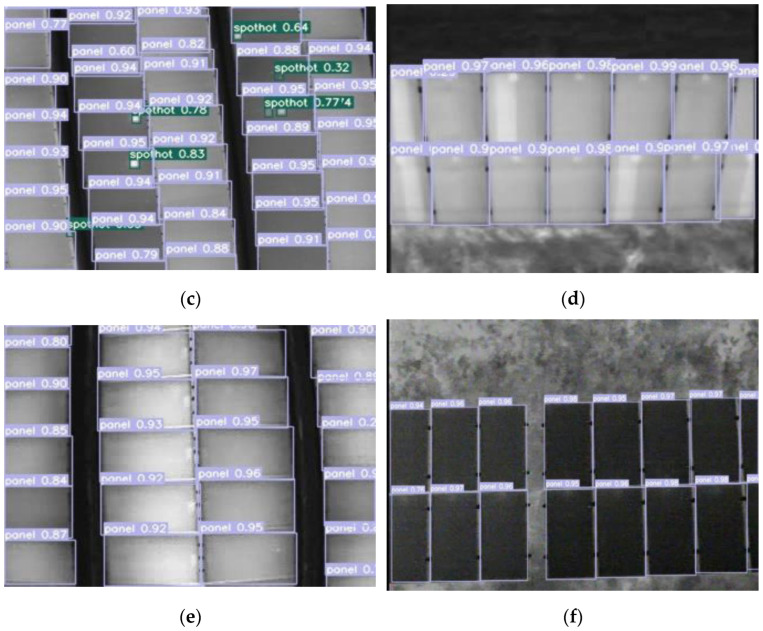
(**a**–**f**) are partial visualization results.

The improved algorithm was used to detect infrared photovoltaic panel images, and the detection effect is shown in Figure 8. The value next to the target box in the figure represents the confidence of labels of different categories. The purple label is the prediction box of photovoltaic panels, and the green label is the prediction box of hot spots.

### 4.4. Comparative Experiments

The comparative experiment not only compared the single-stage object detection algorithms, such as YOLOv3 [37], YOLOv4 [38], and YOLOv5, with different widths and depth, but also compared the two-stage object detection algorithms, such as Faster RCNN [39]. The experimental results are shown in Table 3.

The mAP of the improved algorithm was 98.1%, which was better than the YOLO series algorithm and Faster RCNN. The detection algorithm selected and designed in this study mainly considers its future application environment, i.e., deployment for real-time detection of hot-spot faults. The advantages of the lightweight and high detection speed of the YOLOv5 network can reduce the deployment cost of the recognition model, indicating that the improved model of S-YOLOv5 has great potential for deployment in embedded devices. As can be seen from the experimental results of YOLOv5m, YOLOv5L, and YOLOv5x, under the same network structure, with the increase in neural network layers, the difficulty of small object detection increased, which is not conducive to hot-spot fault detection. Similarly, after several down-sampling iterations, Faster RCNN could not effectively extract features for small targets such as hot-spot fault points, resulting in a reduced detection effect.

In terms of the number of model parameters, the improved lightweight detection model in this paper reduced the number of model parameters by 23.58 times compared with the YOLOv5x algorithm with the maximum depth and width. Furthermore, it is notable that the recognition results of the YOLOv5m algorithm showed that the detection speed of the model was higher than that of the model proposed in this paper. However, the size of the model was relatively large, reaching 21.04 M, which may increase the deployment cost of the recognition algorithm in the embedded devices for picking the robot vision system.

In terms of the speed of model detection, the two-stage Faster RCNN lagged far behind the one-stage object detection algorithms in the detection speed of photovoltaic panels. The FPS of the improved algorithm in this paper reached 49, meeting the requirements of real-time detection.

Overall, the strengths of the proposed detection algorithm are reflected in the following points: firstly, it can automatically recognize the panel and the hot spot in an image; secondly, the detection performance, especially the detection speed and the mAP, of the improved designed S-YOLOv5 is excellent, which is suitable for the real-time hot spot detection; thirdly, the size of the proposed detection model has few parameters and high detection accuracy, indicating that it has great potential to be deployed in hardware devices, which is essential for the wide application of the detection algorithm. A larger model results in higher configuration and computing ability requirements of the hardware equipment.

**Figure 9:**
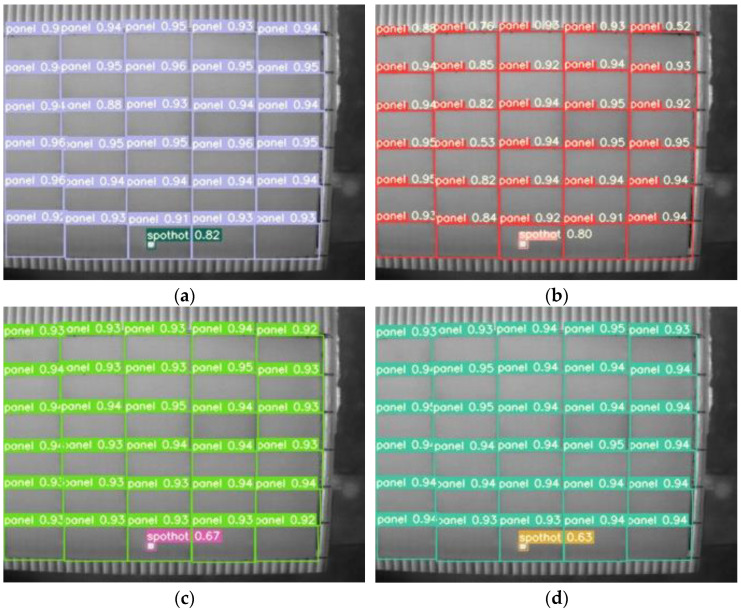

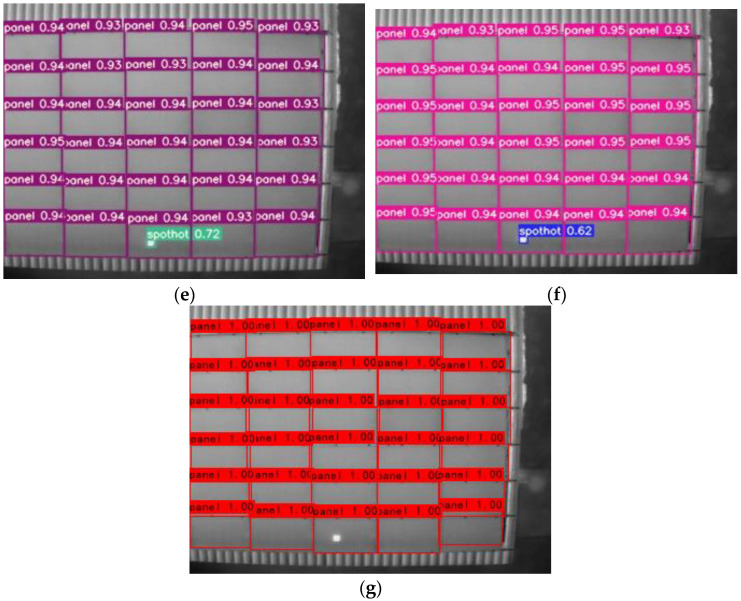
Visualization results from object detection on the same image. (**a**) Our method. (**b**) YOLOv3. (**c**) YOLOv4. (**d**) YOLOv5m. (**e**) YOLOv5l. (**f**) YOLOv5x. (**g**) Faster RCNN.

Figure 9 shows the detection effect of different algorithms on the same infrared photovoltaic panel image. Compared with Faster RCNN, the proposed algorithm detected more targets of photovoltaic panels and hotspots. Compared with YOLOv3, YOLOv4, and YOLOv5m, the improved algorithm in this paper had a higher detection accuracy on hot spots.

## 5. Conclusions

Object detection methods based on convolutional neural networks have been gradually applied in the industrial field and achieved fruitful results. The improved S-YOLOv5 was designed and validated on a real infrared photovoltaic panel image dataset. The Focus structure effectively extracted the features of the input infrared photovoltaic image. Compared with the original network, the lightweight ShuffleNetv2 significantly reduced the number of model parameters. The FPN multiscale fusion method effectively retained the underlying feature information and improved the detection accuracy of hot-spot fault points. The ablation study verified that the improved ShuffleNetv2, Focus, and FPN composite structure had the best hot-spot fault detection effect. Comparative experiments showed that the proposed method has obvious advantages in the number of model parameters and detection speed. In the future, we can try other novel methods to improve the accuracy and efficiency of hot-spot fault detection.

## Figures and Tables

**Table 1:** Infrared photovoltaic image dataset.

Dataset	Pictures	Panels	Hot Spots
Training set	3360	75,102	4990
Validation set	1120	24,760	1560
Test set	1120	24,860	1520

**Table 2:** Ablation study.

Model	Precision (%)	Recall (%)	mAP (%)	Params (M)
Baseline	93.7	92.0	94.4	7.06
+ ShuffleNetv2	92.5	89.9	93.0	0.43
+ Focus + ShuffleNetv2	95.2	94.5	97.0	0.44
+ FPN	94.1	90.7	94.3	6.11
+ ShuffleNetv2 + FPN	94.9	95.6	97.1	3.69
+ Focus + ShuffleNetv2 + FPN	96.0	97.2	98.1	3.71

**Table 3:** Comparative experiments.

Model	mAP (%)	FPS	Params (M)
YOLOv3	96.4	34	61.50
YOLOv4	82.4	44	63.94
YOLOv5m	95.6	54	21.04
YOLOv5l	97.1	46	46.60
YOLOv5x	96.5	37	87.25
Faster RCNN	66.6	7	28.29
Our method	98.1	49	3.71

## Data Availability

Not applicable.
